# Platelet hyaluronidase 2 enrichment in acute coronary syndromes: a conceivable role in monocyte-platelet aggregate formation

**DOI:** 10.1080/14756366.2021.1900159

**Published:** 2021-03-18

**Authors:** Ramona Vinci, Daniela Pedicino, Alessia D’Aiello, Pellegrino Ciampi, Myriana Ponzo, Alice Bonanni, Giulio Russo, Rocco Antonio Montone, Massimo Massetti, Filippo Crea, Giovanna Liuzzo

**Affiliations:** a Catholic University of the Sacred Heart, Rome, Italy; b Fondazione Policlinico Universitario A. Gemelli IRCCS, Rome, Italy

**Keywords:** Acute coronary syndromes, hyaluronic acid, hyaluronidase, platelets, personalised medicine

## Abstract

Acute Coronary Syndromes (ACS) with plaque erosion display dysregulated hyaluronan metabolism, with increased hyaluronidase-2 (HYAL2) expression. However, the expression and the role of this enzyme on platelets has never been explored. We evaluated the platelet’s HYAL2 (_plt_HYAL2) levels on I) stable angina (SA) and II) ACS patients, furtherly sub-grouped in Intact-Fibrous-Cap (IFC) and Ruptured-Fibrous-Cap (RFC), according to Optical Coherence Tomography. We assessed the HYAL2 role through an *in vitro* model setting of co-cultured monocytes and platelets, before and after treatment with low-molecular-weight hyaluronic acid (HA) as pro-inflammatory stimulus and with or without HYAL2-antibody to inhibit HYAL2 activity. ACS patients exhibit higher _plt_HYAL2 levels comparing to SA, with the higher expression for IFC group. The addition of HYAL2-antibody significantly reduced the percentage of monocyte-platelet binding, suggesting that _plt_HYAL2 enrichment at the site of the culprit lesion is a key mediator in the systemic thrombo-inflammatory status of ACS presenting with plaque erosion.

## Graphical Abstract

**Figure:**
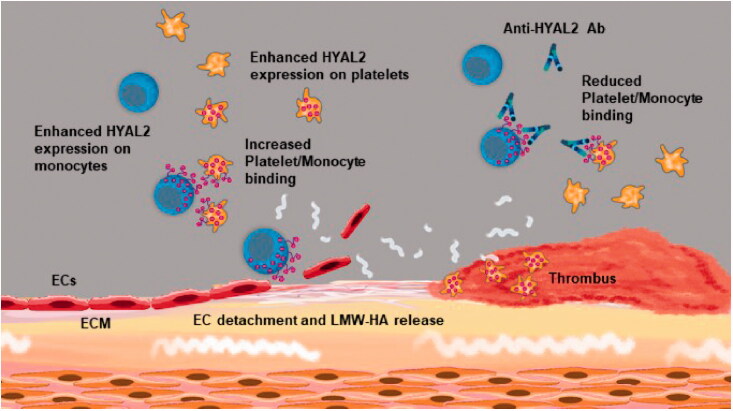

Representation of platelet Hyaluronidase 2 involvement in monocyte-platelet aggregates and pathogenesis of plaque erosion. (Ab, antibody; ECM, extracellular matrix; EC, endothelial cells, HYAL2, hyaluronidase 2; LMW-HA, low molecular weight-hyaluronan)

## 1. Introduction

Hyaluronic acid (HA) is an ubiquitous, natural, anionic glycosaminoglycan (GAG) of repeating β1,4-N-acetyl-D-glucosamine and β1,3-D-glucuronic acid units^1^ with a biological function strictly dependent on its molecular weight^2^. The high molecular weight HA polymers (HMW-HA; MW >1000 kDa), physiological key components of vascular extra cellular matrix (ECM), maintain tissue homeostasis promoting anti-inflammatory and tissue repairing processes, while the low molecular weight counterparts (LMW-HA; MW <250 kDa) are involved in pro-inflammatory biological pathways and ECM derangement^2^.

Acute Coronary Syndrome (ACS) patients display dysregulated HA metabolism^3^^,^^4^ that furthermore might promote the generation of monocyte-platelet complexes^4^, involved in increased platelet reactivity and in thrombus formation^5^. Systemic blood mononuclear cells from ACS presenting with Intact Fibrous Cap (IFC) atherosclerotic plaque at Optical Coherence Tomography (OCT) show increased levels of Hyaluronidase 2 (HYAL2), the key enzyme responsible of HA hydrolysis and LMW-HA generation^4^. Alongside mononuclear cells, platelets can bind HA as well; indeed, platelets and their precursors express HYAL2 and can stimulate mononuclear leukocytes to produce pro-inflammatory mediators^6^.

Aims of this study were to assess HYAL2 protein expression on platelets (_plt_HYAL2) of patients presenting with ACS and to understand if the augmented cross-talk between platelets and immune cells depends on HYAL2, as a consequence of an altered HA metabolism.

## 2. Materials and methods

### 2.1. Population study

We enrolled (1) patients with symptoms of stable effort angina (SA) (*n* = 40) lasting more than 1 years, angiographically confirmed coronary artery disease, with no prior acute coronary events, and no ischaemic episodes during the last 48 h; (2) ACS patients at their first diagnosis admitted to our coronary care unit with non–ST-segment elevation myocardial infarction (NSTEMI) (*n* = 40). The NSTEMI group was furtherly divided in two sub-groups, according to the Optical Coherence Tomography (OCT) analysis of the culprit plaque during coronary angiography: Intact Fibrous Cap (IFC) group and Ruptured Fibrous Cap (RFC) one.

All patients gave their written informed consent and the Ethics Committee of the Fondazione Policlinico Universitario “Agostino Gemelli” IRCCS–Università Cattolica del Sacro Cuore (UCSC) approved the study (Prt. N. 37077/19).

### 2.2. Platelet isolation and multicolour flow-cytometry analyses

Cellular analyses were performed on platelets isolated from peripheral whole blood collected in citrate dextrose tubes as follows. Platelet rich plasma (PRP) was obtained by centrifugation of fresh blood at 200 x g for 10 min at room temperature (RT). PRP was transferred into a new plastic tube, without disturbing the buffy coat layer, and centrifuged as above for 5 min to remove the residual erythrocytes/white blood cells. PRP supernatant was withdrawn and centrifuged in Acid-Citrate-Dextrose (ACD) solution (1 part ACD solution to 9 parts blood) (Sigma-Aldrich) at 800 x g for 20 min at RT. Platelet pellets were gently washed and resuspended in Tyrode’s buffer (pH 7.8) and aliquoted according to the final analyses.

We performed a multicolour flow-cytometry for evaluating the surface protein expression of HYAL2 by staining isolated platelets with unconjugated HYAL2 antibody (ThermoFisher Scientific). The secondary antibody for the HYAL2 was Alexa Fluor 488-conjugated goat anti-rabbit IgG (1:1000) (Invitrogen).We used CD45-ECD antibody (Beckman Coulter) for verifying the purity of our platelet samples and anti-CD62P-PE antibody (eBioscience, INC.) to determine platelet activation.

### 2.3. _plt_HYAL2 expression according to OCT investigation of coronary artery stenosis

We compared _plt_HYAL2 expression, as described above, within NSTEMI patients by sub-grouping them according to OCT investigation (C7-XR or ILUMIEN OPTIS, St. Jude Medical) in IFC plaques (*n* = 6) and Ruptured Fibrous Cap (RFC) plaques (*n* = 8). We classified as plaque erosion the presence of thrombus overlying a plaque with IFC, or the presence of luminal surface irregularity at the culprit lesion in the absence of thrombus, and as RFC the presence of fibrous cap discontinuity with a cavity formed inside the plaque or with a direct communication between lumen and inner core of a plaque^7^.

### 2.4. Isolation of human peripheral mononuclear cells

We isolated peripheral blood mononuclear cells (PBMCs) from whole blood Ethylene Diamine Tetraacetic Acid (EDTA) samples by density gradient centrifugation method (Lympholyte®-H Cell Separation Media, CEDARLANE). PBMC pellets were washed and resuspended in Dulbecco’s phosphate-buffered saline (DPBS) (GIBCO, Invitrogen) and aliquoted according to the final analyses.

### 2.5. Hyal2 inhibition experiment using an *in vitro* monocyte-platelet model

We investigated the HYAL2 role through an *in vitro* model setting of co-cultured monocytes and platelets. Co-coltures were incubated for 16 h at 37 °C under 5% CO2 and 20% O2 (humidified incubator), in RPMI 1640 medium (LONZA) supplemented with 100 U/mL penicillin, 0.1 mg/mL streptomycin, 2 mM glutamine and 10% foetal bovine serum (Thermo-Fisher) as follows not treated (NT) or treated with HMW- (1350 kDa, 100 µg/mL) and LMW- HA (MW 20 kDa, 100 µg/mL) (R&D systems by Lifecore Biomedical, LLC), and *Escherichia Coli*-lipopolysaccharide (1 µg/mL; LPS; Sigma-Aldrich), before and after inhibition of HYAL2 through HYAL2 antibody treatment (2 µg/mL; Abcam). The immunogen used in generating the anti-HYAL2 is a keyhole limpet haemocyanin conjugated synthetic peptide between 1 and 100 amino acids from Human HYAL2.

### 2.6. Flow-cytometry analyses of cultured cells

We assessed the monocyte-platelet binding by analysing the frequency of CD14-CD42 positive cells (% CD14^+^CD42^+^) after staining with CD14-ECD antibody (Beckman Coulter) as monocyte marker, and CD42b-FITC antibody (Beckman Coulter) as platelet marker.

### 2.7. Enzyme-Linked immunosorbent assay (ELISA) of serum HA

We checked the circulating levels of total HA content performing an Enzyme-Linked Immunosorbent Assay (Hyaluronan Quantikine ELISA Kit, R&D Systems) on sera collected from SA patients and NSTEMI patients undergone to OCT, according to the manufacturer’s recommendation.

### 2.8. Statistical analyses

Distribution of variables (described as Mean ± SD) were assessed by Shapiro Wilk test. We performed unpaired T-test and Mann-Whitney test (for nonparametric variables) to compare two groups, as appropriate. ANOVA for repeated measures, with Bonferroni’s test were used for multiple comparisons. For all the experimental assays performed, a two-tailed *p* values ≤ 0.05 was considered statistically significant. We used multicolour flow-cytometry and Kaluza software for protein detection and analysis (FC 500 and Kaluza Software, Beckman Coulter, Brea, C.) and GraphPad Prism 8 Software (GraphPad Software, Inc., San Diego, CA, USA) for data analysis.

## 3. Results and discussion

**Figure 1.**
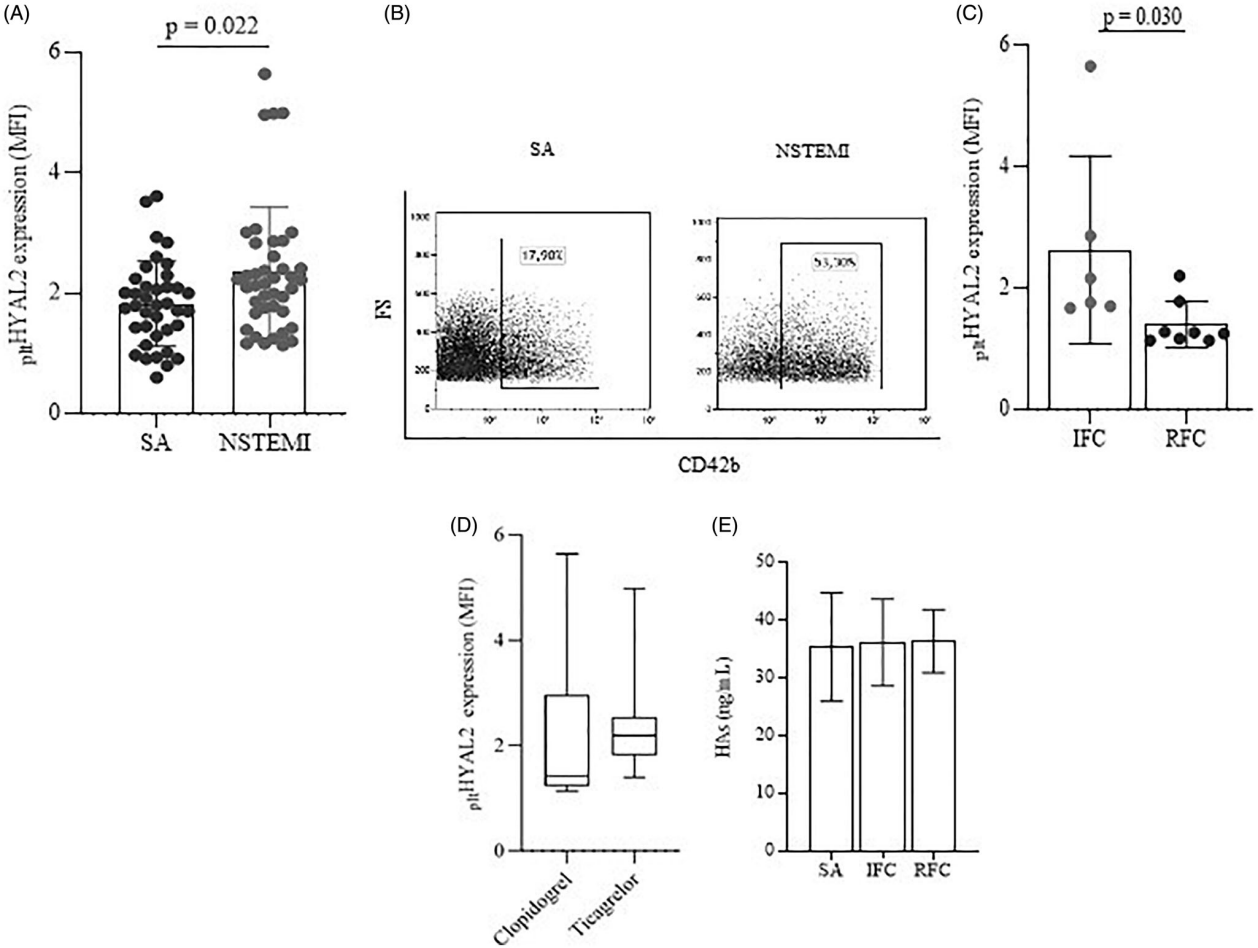
Platelet Hyaluronidase 2 expression in ACS patients presenting with stable and unstable plaques. (A) _plt_HYAL2 protein surface expression of SA and NSTEMI patients. (B) Representative flow-cytometry dot plots showing platelet HYAL2^+^ and their frequency on an SA (left) and NSTEMI (right) patients. (C) _plt_HYAL2 protein surface expression according to OCT evaluation (values are presented as mean ± standard deviation (SD). (D) _plt_HYAL2 protein surface expression of NSTEMI patients according to anti-platelet therapies. (E) HA circulating levels (CD, cluster of differentiation; FS, forward scatter; IFC, intact fibrous cap; MFI, mean fluorescence intensity; NSTEMI, non–ST-segment elevation myocardial infarction; OCT: optical coherence tomography; RFC: ruptured fibrous cap; SA: stable angina).

The _plt_HYAL2-MFI analysis (Figure 1(A and B)) assessed that NSTEMI patients had a significant increase of _plt_HYAL2 expression with respect to SA patients (2.34 ± 1.01 vs 1.83 ± 0.71, respectively; *p* = 0.022) (Figure 1(A)). Analyses of the NSTEMI sub-groups, according to OCT investigation, revealed an increased expression of _plt_HYAL2 in patients with IFC plaques compared to those ones with RFC plaques (2.63 ± 1.50 vs 1.40 ± 0.40, respectively; *p* = 0.030) (Figure 1(C)). These results demonstrated, for the first time, that patients presenting with unstable plaques such as NSTEMI patients have a significant systemic increase of _plt_HYAL2 when compared to those ones with stable atherosclerotic lesions, and that, interestingly, this different expression is significantly driven by patients with IFC lesions.

A drug-depending analysis showed that anti-platelet therapies with clopidogrel or ticagrelor did not affect _plt_HYAL2 expression (Figure 1(D)) underling that _plt_HYAL2 expression seems to have a role independent of common treatment with P2Y12 receptor inhibitors, thus suggesting an alternative signalling route.

HA serum circulating levels were similar between SA patients and patients with IFC and RFC plaques (Figure 1(E)), remaining around the normal human concentration^8^. Since HA has a pathophysiological role that is strictly related to its molecular weight^2^, an explanation might be that the assay does not detect HA molecules with a size lower than 35 kDa, thereby excluding a relevant amount of hydrolysed glycosaminoglycan content.

**Figure 2.**
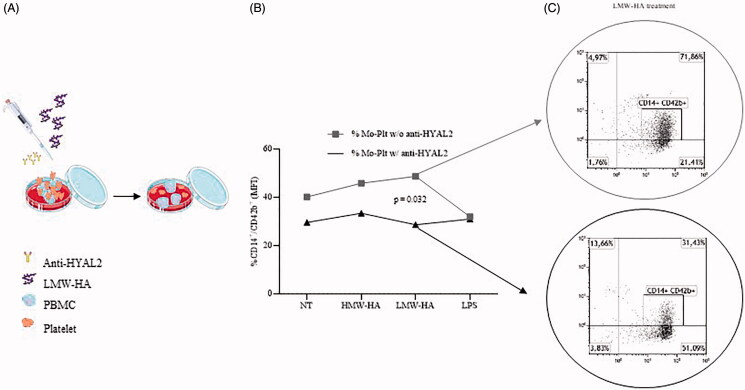
Hyaluronic acid catabolism involvement in monocyte-platelet binding. (A) Schematic representation of the *in vitro* experiments (art images from http://smart.servier.com/ site). (B) Modulation of monocyte-platelet binding in cells isolated from NSTEMI patients and treated with three different stimuli before and after anti-HYAL2 incubation. (C) Representative flow-cytometry images showing modulation of monocyte-platelet binding in cells isolated from NSTEMI patients and treated with LMW-HA before and after anti-HYAL2 incubation (CD, cluster of differentiation; HYAL2, hyaluronidase 2; HMW-HA, high molecular weight-hyaluronan; LMW-HA, low molecular weight-hyaluronan; LPS, lipopolysaccharide; NT, not treated; w/, with; w/o, without).

Moreover, in NSTEMI patients, our *in vitro* model (Figure 2(A)) showed a significant reduction of monocyte-platelet binding (% CD14^+^CD42^+^) following the incubation with anti-HYAL2 antibody, only for LMW-HA treated samples (*p* = 0.032; Figure 2(B and C)). These *in vitro* setting corroborated that the synergic interaction between monocytes and platelets might play a role in ECM derangement and plaque destabilisation especially in presence of an altered HA catabolism, and that the HYAL2 surface ligation may negatively affect the monocyte-platelet aggregate formation.

Human platelets, whose count is significantly associated to all cause of mortality in patients with myocardial infarction, express bacterial receptors like TLRs that could trigger the thrombo-inflammatory response through activation of phosphoinositide 3-kinase^9–11^. Indeed, as a direct consequence of plaque destabilization, platelets leave their quiescent state, adhere to the injured vascular endothelium and actively interact with the extracellular matrix (ECM), by releasing its α−granule content, including HYAL2 molecules^12^. Alongside, the innate immune system appears to be involved in ECM derangement and HA dyshomeostasis^13^ through the commitment of molecule such as monocyte HYAL2^4^ or HA synthase 3 (HAS3)^14^.

Thus, if on one side the profound perturbation of the immune system together with its residual inflammatory *milieu* might describe the mechanisms of plaque rupture^15^^,^^16^, on the opposite hand, the dysregulation of HA metabolism in ECM might explain the pathogenesis of plaque erosion (Graphical Abstract).

Clinicians should refine the current pharmacological treatments bearing in mind the different biological mechanisms underlying plaque erosion and plaque rupture^17^^,^^18^ and our results, in concert with those ones obtained on systemic mononuclear cells^4^, might open the way for a novel and increasingly personalised medicine to supplement the classical anti-thrombotic therapies in patients with myocardial infarction.

## Conclusions

HYAL2 might be the bridge between elements historically belonging to the immune system, such as monocytes, and elements belonging to the haemostasis and thrombotic system, such as platelets, representing a novel target to be reached in view of therapies increasingly personalised.
